# Repetitive transcranial magnetic stimulation for treatment of limb spasticity following multiple sclerosis: a systematic review and meta-analysis

**DOI:** 10.1590/1414-431X2023e12708

**Published:** 2023-05-29

**Authors:** Dongyun Su, Anzi Wang, Meirong Zhu, Fei Yang, Wei Li, Bo Ma, Min Liu, Zongqi Li, Bo Wang, Huanyi Tu, Bo Ning

**Affiliations:** 1 Department of Neurology, The First People's Hospital of Longquanyi District Chengdu, Longquanyi District, Chengdu, China; 2 Department of Hematology, The Third People's Hospital of Chengdu, Qingyang District, Chengdu, China

**Keywords:** Efficacy, Meta-analysis, Multiple sclerosis, Spasticity, Repetitive transcranial magnetic stimulation

## Abstract

Pilot trials have suggested that repetitive transcranial magnetic stimulation (rTMS) may reduce limb spasticity in multiple sclerosis (MS). We carried out the current meta-analysis to synthesize currently available evidence regarding such correlation. Up to November 2022, five international electronic databases (Cochrane CENTRAL, PubMed, Embase, Web of Science, and CINAHL) and four Chinese electronic databases (CBM, CNKI, WanFang Data, and VIP) were systematically searched to identify randomized trials comparing active rTMS and sham stimulation in patients with MS-related spasticity. Two reviewers independently selected studies and extracted data on study design, quality, clinical outcomes, and time points measured. The primary outcome was clinical spasticity relief after intervention. Secondary outcomes included spasticity at the follow-up visit 2 weeks later and post-treatment fatigue. Of 831 titles found, we included 8 studies (181 participants) in the quantitative analysis. Pooled analyses showed that rTMS therapy was associated with significant spasticity relief in the early post-intervention period [standardized mean differences (SMD): -0.67; 95%CI: -1.12 to -0.21], but there was insufficient evidence for rTMS in reducing spasticity at the follow-up visit 2 weeks later (SMD: -0.17; 95%CI: -0.52 to 0.17) and fatigue (SMD: -0.26; 95%CI: -0.84 to 0.31). This evidence supports the recommendations to treat MS-related spasticity with rTMS, but underlines the need for further large randomized trials.

## Introduction

Multiple sclerosis (MS) is a chronic neurological and inflammatory disorder of the central nervous system (CNS) that predominantly occurs in young adults and affects approximately 2.5 million people worldwide (1-3). MS occurs in early life and has only a modest negative effect on longevity, so that problems of rapidly progressing physical disability, activity restriction, social isolation, and psychosocial adjustment progress over time and place a significant burden on affected individuals, informal caregivers, and the health care system (4,5). Spasticity is a common disabling physical symptom in patients with MS, with reports of affecting 60-90% of MS survivors (6-8). It may affect the trunk musculature, resulting in restricted joint mobility, poor postural control, and loss of dexterity, impacting caregiver burden and quality of life (QoL) (9). Furthermore, spasticity has a direct correlation with disease progression and other condition-related impairments such as pain, fatigue, and cognitive deficits, which also impact patient function (10).

Pharmacological and non-pharmacological interventions are the mainstay of spasticity management for MS patients. The former includes antispasmodic medications such as baclofen, benzodiazepines, dantrolene sodium, and tizanidine, while the latter includes physical, surgical, and instrumental approaches. However, current medical treatments are partially effective and can be associated with various systemic side effects (drowsiness, muscle weakness, and cognitive impairment) (11,12). Neurosurgical procedures are invasive and considered only for severe spasticity following the failure of pharmacological agents (13). Therefore, new approaches for MS treatment are needed.

Repetitive transcranial magnetic stimulation (rTMS) is a neurostimulation and neuromodulation technique that has been widely applied to treat several symptoms in people living with MS (pwMS) (14). Due to its neuroplasticity properties and effects on motor cortex excitability at both short- and long-lasting intervals, rTMS can modulate motor corticospinal output, thereby remodeling local and distant excitability with a tangible effect on limb spasticity (15-17). Current data regarding the relationship between application of rTMS over the primary motor cortex and symptoms of limb spasticity in pwMS are conflicting. Some authors have reported reduction of spasticity associated with high-frequency rTMS (HF-rTMS) and intermittent theta-burts (iTBS) treatment, but these findings have not been consistently replicated (18-25). A recent meta-analysis (26), which identified 8 original controlled studies that directly compared active rTMS and sham stimulation totaling 161 MS patients, failed to observe significant anti-spastic effects of active rTMS. However, retrospective checks confirmed that one eligible trial (24) had been omitted in the process of literature screening. Given that the omission of relevant data probably introduced bias and affected the reliability of relevant results, no formal recommendation could be made for this indication. Furthermore, no prior meta-analyses have focused exclusively on patients diagnosed of MS-related spasticity to date. The aim of this work was to evaluate the intervention effects of rTMS in pwMS with spasticity through a systematic review and meta-analysis of evidence from randomized controlled trials.

## Material and Methods

### Data sources and search strategy

We searched multiple international cross-disciplinary electronic databases (PubMed, Embase, Web of Science, Cochrane CENTRAL, and CINAHL) and Chinese electronic databases (Chinese Bio-medicine Database [CBM], WanFang Data, Chinese National Knowledge Infrastructure [CNKI], and China Science and Technology Journal Database databases [VIP]) from inception of each database until November 2022. The selected keywords were obtained by review of primary search results and controlled vocabularies - Medical Subject Headings (MeSH) in PubMed and Cochrane library (Supplementary Table S1 for details). Searches were limited to completed human studies, published in English or Chinese, in any year. Bibliographies of journal articles, reviews, and editorials were manually searched to identify potentially eligible studies. We also reviewed abstracts from major international meetings in the field to locate any unpublished studies. This work was conducted according to the Preferred Reporting Items for Systematic Reviews and Meta-Analyses (PRISMA) guidelines (27) and was registered in PROSPERO (International Prospective Register of Systematic Reviews; reference number: CRD42022352922).

### Study selection

After removing the duplicates, the 2 principal investigators (D.Y.S. and A.Z.W.) independently screened the title and abstract of retrieved studies according to pre-specified inclusion/exclusion criteria. In case of uncertainty, the full texts of the selected studies were reviewed. Any disagreement on the process of selection was resolved by consensus.

Studies were included if they met the following criteria: i) participants had a confirmed diagnosis of MS based on validated criteria and suffered from limb spasticity as measured by the Modified Ashworth Scale (MAS) scored greater than or equal to one point; ii) randomized controlled trials (RCTs) comparing real rTMS with sham control. Trials performing less than 10 intervention sessions were excluded. The frequency of stimulation, target area, intensity, and duration were not limited; iii) at least one clinical outcome was reported for spasticity or fatigue, irrespective of whether it was a primary or secondary outcome. We excluded non-randomized observational studies, reviews, comments, and trials that did not provide sufficient data. If multiple investigations were based on the same population data, the articles with comprehensive information would be included.

### Data extraction and quality assessment

The articles that met the inclusion criteria were obtained in electronic format. Two investigators (D.Y.S. and A.Z.W.) independently extracted data from eligible papers on standardized Microsoft Excel spreadsheets, based on the Cochrane data extraction tool. Data extraction forms were used to collect pertinent information about study type, the first author, year of publication, country, study inclusion and exclusion criteria, number of patients in each treatment arm, type of MS, number of pulses per session, intensity of stimulation, risk of bias, details on rTMS and comparator groups, outcomes, and time points measured. We attempted to contact the authors of primary studies to supplement incomplete information. Assessment of the risk of bias in included studies was performed according to the Risk of Bias Assessment Tool from the Cochrane Handbook (28), and any discrepancy or uncertainty was resolved by consensus among authors.

### Definition of outcomes

The primary outcome was defined as short-term (within 1 week after the last session) rTMS effect as measured by mean score changes in clinical spasticity symptoms from baseline to end-point. If data were available for more than one time-point in the article, we gave preference to the time-point closest to the last session. If a study used multiple spasticity measures, preference was given to the measure reported by the majority of the included studies. If a study reported two or more outcome measures, and none of the other included studies used any of them, preference was given to the measure listed as primary in this study.

Pre-specified secondary analyses included the following: i) efficacy at short-term follow-up, as measured by mean score change in spasticity symptoms from baseline to 2 weeks after the end of the stimulation course. The selection priority of spasticity scales was the same as for the primary outcome; ii) fatigue symptoms, as measured by mean score change on fatigue severity scales from baseline to just after the last session.

### Data synthesis and analysis

To pool data from different continuous scales and ordinal scales with no standard cut-off point, we calculated standardized mean differences (SMD) and 95% confidence intervals (CIs) for the same outcomes. Between-study heterogeneity was evaluated using the Cochran Q test and I^2^ value. When I^2^>40% and P value <0.1, pooled risk estimates were calculated using the DerSimonian and Laird random-effects models, adjusting for within-study and between-study variation. The fixed-effects (Mantel-Haenszel) models were used to obtain more precise estimates where there was no evidence of significant heterogeneity (29). Subgroup analyses with regard to the primary outcome were performed based on type of MS (relapsing remitting *vs* secondary progressive), type of intervention (HF-rTMS *vs* iTBS), sex ratio (female/male, >1 *vs* ≤1), and whether it was combined with conventional rehabilitation to explore the effect of these indicators on spasticity symptom in pwMS. Sensitivity analyses were performed by sequentially removing individual studies to assess whether any specific report could influence the overall results. In addition to funnel plots, the Egger test (30) was performed for publication bias assessment. All statistical analyses were performed using Review Manager 5.3.3 (Cochrane, UK) and Stata 12 (USA) software, with a P value of less than 0.05 considered statistically significant.

## Results

### Search results and characteristics of included studies

**Figure 1:**
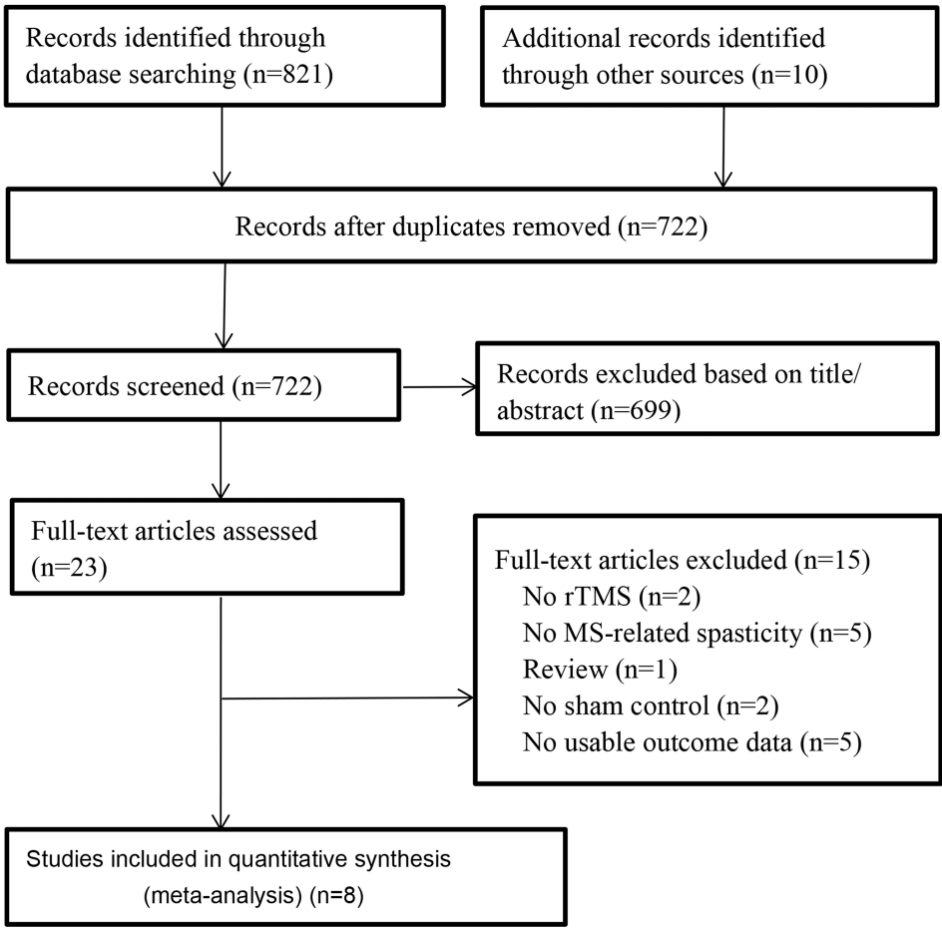
Flow diagram of literature search and study selection. RCT: randomized controlled trial; MS: multiple sclerosis; rTMS: repetitive transcranial magnetic stimulation.

The process of study selection is shown in Figure 1. Our search identified 831 potentially eligible citations (Supplementary Table S2). After removing the duplicates and review of titles and abstracts, 699 citations were excluded and 23 were ultimately retained for further evaluation. Fifteen studies were excluded for the following reasons: not rTMS (n=2), not MS-related spasticity (n=5), review articles (n=1), not sham control (n=2), or lack of pertinent data (n=5).

The design of 8 studies included (18-25) in this meta-analysis are summarized in Table 1. The studies were published from 1996 to 2019 and were conducted in 6 countries. Among 181 patients diagnosed with MS-related spasticity in 8 randomized trials, 106 patients were assigned to HF-rTMS or iTBS, and 75 patients were assigned to sham stimulation, with a mean age of 50 years and a sex ratio of 0.7 (male/female 75/106). Four studies involved patients with relapsing remitting (RR) MS (19,20,22,23), one included patients with secondary progressive (SP) MS (21), one recruited both RR and SP MS (18), and the remaining two studies did not specify the type of MS (24,25). In addition, there were 5 studies that concerned conventional limb rehabilitation (such as physical therapy or exercise therapy). For those studies that had more than one active group, we considered each arm as one study in the quantitative analysis.

**Table 1:** Characteristics of included randomized controlled trials.

Reference, year	Country	Type of disease	Intervention		Sample size (T/C), n	Mean age (year)	Gender (female, %)	Intensity	No. of pulses	Assessments	Main findings
			Treatment group	Comparator group							
Boutiàre 2016 18	France	SP/RR	iTBS once daily for 13 consecutive working days + standardized rehabilitation	Sham iTBS + standardized rehabilitation	9/8	51.6±10.6	47.06%	80% AMT	600	VAS	Improvement of spasticity was greater in iTBS group than in sham iTBS group (P=0.026)
Centonze 2007 19	Italy	RR	5 Hz rTMS once daily for 5 consecutive days for 2 weeks	Sham rTMS	12/7	41.4±6.1	73.68%	100% RMT	900	MAS	rTMS may improve spasticity in MS
Dieguez-Varela 2019 20	Spain	RR	iTBS once daily for 10 treatment sessions (two weeks)	Sham iTBS	10/7	49.8±9.8	58.82%	80% AMT	600	MAS	iTBS did not produce any significant clinical effect on spasticity
Korzhova 2019 21	Russia	SP	20 Hz rTMS once daily for 5 consecutive days for 2 weeks + physical therapy	Sham rTMS + physical therapy	12/5	41.7±17.7	61.76%	80% AMT	1600	MAS, SESS, NAS, MFIS	MAS was significantly reduced after the stimulation course in the HF-rTMS group
Korzhova 2019 21	Russia	SP	iTBS once daily for 5 consecutive days for 2 weeks + physical therapy	Sham iTBS + physical therapy	12/5	46.7±7.8	55.88%	80% MSP	1200	MAS, SESS, NAS, MFIS	MAS was significantly reduced after the stimulation course in the iTBS group
Mori 2010 22	Italy	RR	iTBS once daily for 5 consecutive days for 2 weeks	Sham iTBS	10/10	44.3±12.5	65%	80% AMT	600	MAS	Patients receiving iTBS showed a significant reduction of MAS scores
Mori 2011 23	Italy	RR	iTBS once daily for 5 consecutive days for 2 weeks + exercise therapy	Sham iTBS + exercise therapy	10/10	38.4±11.2	35%	80% AMT	600	MAS, MSSS-88, FSS	A combination of iTBS and ET ameliorated spasticity
Nielsen 1996 24	Denmark	NA	25 Hz rTMS twice daily for 7 consecutive days + daily physical activity and physical therapy	Sham rTMS + daily physical activity and physical therapy	21/17	44 (26-67)	68.42%	NA	NA	Clinical spasticity score	rTMS had an antispastic effect in MS
Şan 2019 25	Turkey	NA	5 Hz rTMS once daily for 10 sessions lasting 2 weeks + physical therapy and rehabilitation program	Sham rTMS + physical therapy and rehabilitation program	10/6	49.93±12.27	50%	110% RMT	900	MAS	Active rTMS reduced spasticity in MS patients compared to control group

AMT: active motor threshold; FSS: Fatigue Severity scale; iTBS: Intermittent theta burst stimulation; MAS: Modified Ashworth scale; MFIS: Modified Fatigue Impact scale; MS: multiple sclerosis; MSP: maximum stimulant power; MSSS-88: 88 items Multiple Sclerosis Spasticity Score questionnaire; NA: not available; NAS: Numerical Analog scale; RMT: resting motor threshold; RR: relapsing remitting; rTMS: repetitive transcranial magnetic stimulation; SESS: Subjective Evaluating Spasticity scale; SP: secondary progressive; T/C: treatment/comparator; VAS: Visual Analogue scale.

### Outcomes

**Figure 2:**
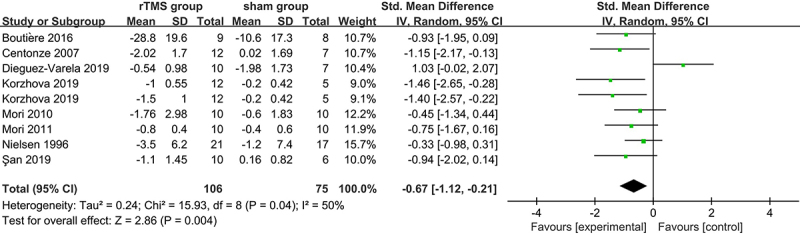
Forest plot for short-term relief of spasticity symptoms. CI: confidence interval; df: degrees of freedom; IV: inverse variance; SD: standard deviation; Std: standardized; rTMS: repetitive transcranial magnetic stimulation. See Table 1 for reference numbers.

**Figure 3:**
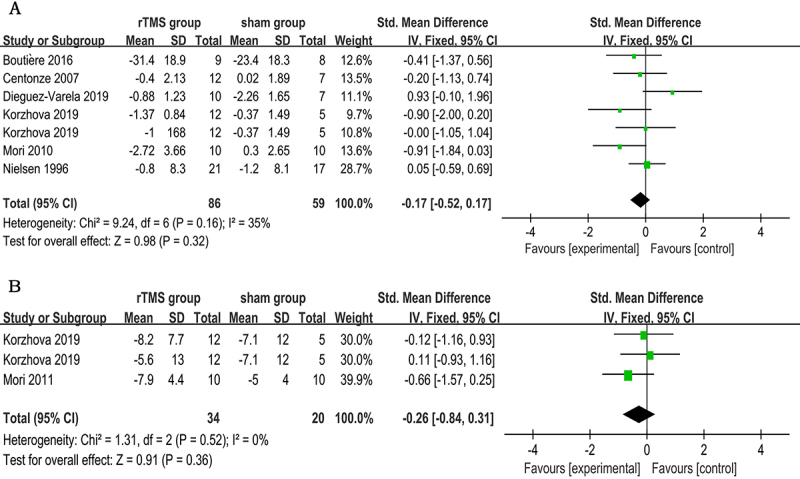
Forest plot of secondary outcomes. **A**, Spasticity at the follow-up visit 2 weeks later. **B**, Post-treatment fatigue. CI: confidence interval; df: degrees of freedom; IV: inverse variance; SD: standard deviation; Std: standardized; rTMS: repetitive transcranial magnetic stimulation. See Table 1 for reference numbers.

Eight trials totaling 181 patients provided data sufficient for meta-analysis on the short-term spasticity outcome after treatment. There was significant heterogeneity among the studies (P=0.04, I^2^=50%), thereby random-effects models were used for analysis. The overall pooled effect size calculated using the DerSimonian-Laird method showed a significant benefit of rTMS therapy in reducing limb spasticity within a week of the last session (SMD: -0.67; 95%CI: -1.12 to -0.21; P=0.004; Figure 2). As for spasticity reduction at the follow-up visit 2 weeks later, there was no statistical difference between active rTMS group and sham stimulation group (SMD: -0.17; 95%CI: -0.52 to 0.17; P=0.32; I^2^=35%; Figure 3A). In terms of fatigue outcome, data were pooled and calculated using fixed-effects models. No statistical difference was observed between these two groups (3 trials, n=54; SMD: -0.26; 95%CI: -0.84 to 0.31; P=0.36; I^2^=0%; Figure 3B).

### Subgroup analyses

**Figure 4:**
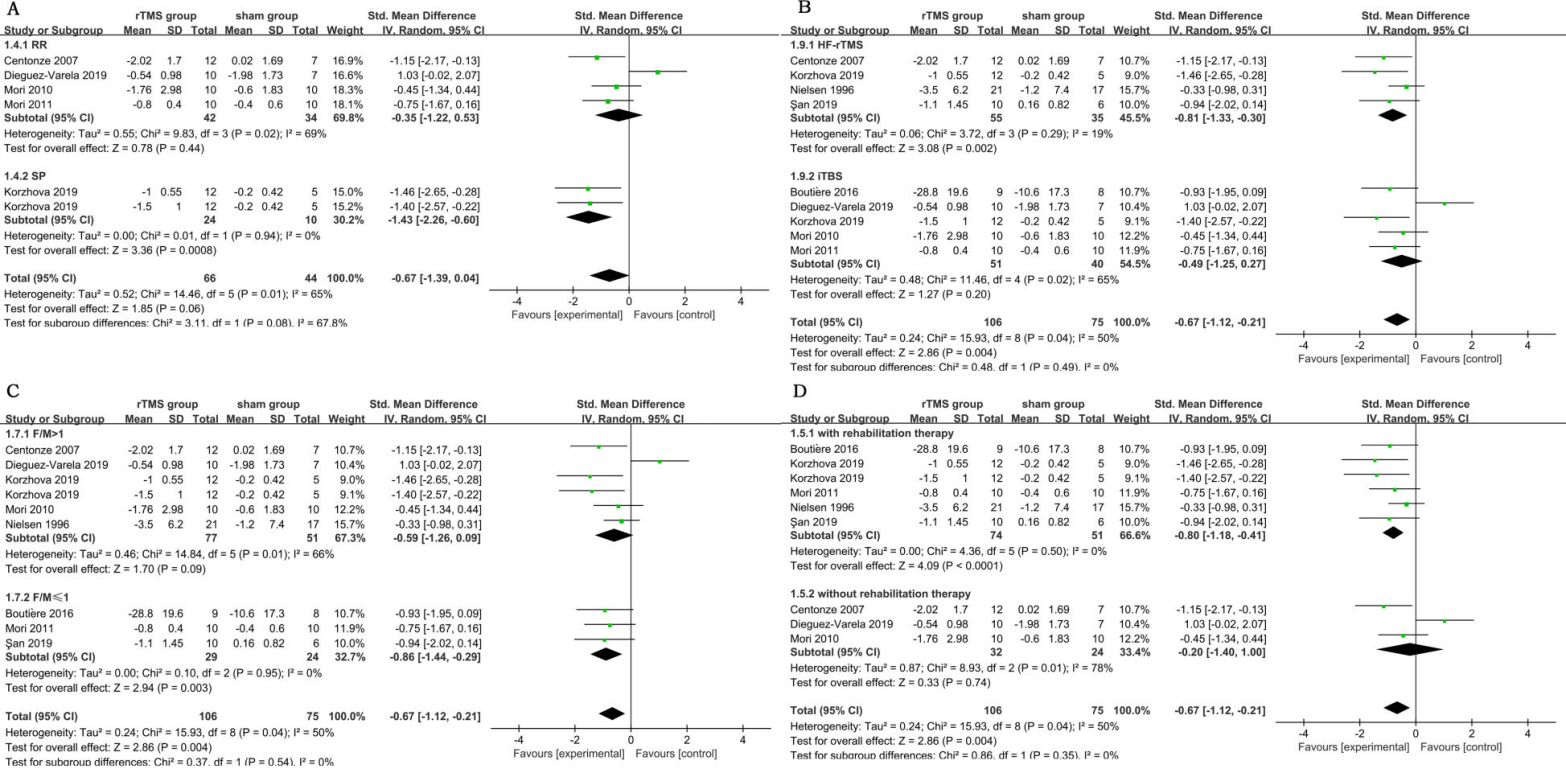
Standardized mean difference in change in spasticity scores from baseline between the rTMS and control groups, stratified by (**A**) type of multiple sclerosis (MS), (**B**) type of intervention, (**C**) sex ratio, and (**D**) whether combined with conventional rehabilitation. CI: confidence interval; df: degrees of freedom; IV: inverse variance; SD: standard deviation; Std: standardized; rTMS: repetitive transcranial magnetic stimulation. See Table 1 for reference numbers.

When stratified by type of MS, rTMS therapy significantly reduced limb spasticity among SP MS (SMD: -1.43; 95%CI: -2.26 to -0.60) but not RR MS (SMD: -0.35; 95%CI: -1.22 to 0.53); however, the difference between the two subgroups was not significant (P for interaction=0.08) (Figure 4). In a subgroup analysis by type of intervention, there was no difference between iTBS (SMD: -0.49; 95%CI: -1.25 to 0.27) and HF-rTMS (SMD: -0.81; 95%CI: -1.33 to -0.30) concerning the spasticity outcome (P for interaction=0.49). Similarly, in a subgroup analysis based on sex, no significant subgroup difference was observed (P for interaction=0.54). In addition, a subgroup analysis was also performed according to whether treatment was combined with conventional rehabilitation. A larger effect size was observed when rTMS application was combined with rehabilitation training (SMD: -0.80; 95%CI: -1.18 to -0.41 *vs* SMD: -0.20; 95%CI: -1.40 to 1.00); however, the difference between the two subgroups was not significant (P for interaction=0.35).

### Sensitivity analyses

Sensitivity analyses were performed to examine the robustness of our results. In order to identify whether any research had a disproportionate influence on the summary treatment effect, we removed studies one at a time. Deleting individual studies did not significantly alter the results. In the study by Şan et al. (25), the evaluation parameters of short-term effects were assessed one week after the last session rather than immediately after applying rTMS. Sensitivity analysis by excluding this study achieved a consistent result (Supplementary Figure S1).

### Quality assessment and publication bias

**Figure 5:**
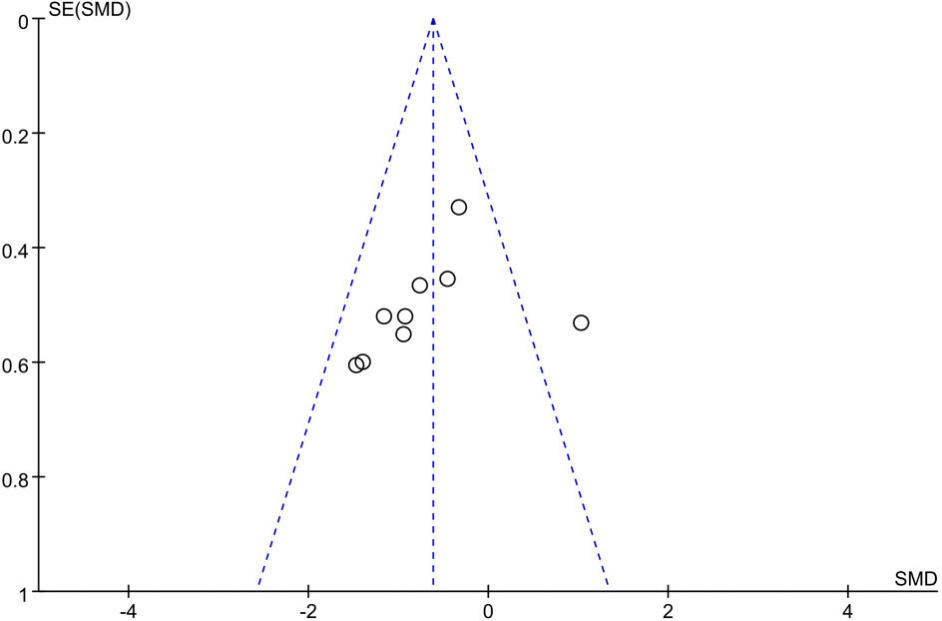
Funnel plot for publication bias for primary outcome. SE: standard error; SMD: standardized mean difference.

The tool for assessing risk of bias recommended by the Cochrane Collaboration was used to assess the quality of the included RCTs (Supplementary Figure S2). The overall bias was low in 3 studies (19,22,24) and medium in 5 studies (18,20,21,23,25). The inverted funnel plot for motor outcome was visually asymmetric (Figure 5). Egger regression test indicated no publication bias (P=0.319), but Begg rank correlation test indicated possible publication bias (P=0.029) (Supplementary Figure S3). The trim-and-fill method was used to recalculate our pooled risk estimate. The analysis showed that the imputed effect size was -0.71 (95%CI: -1.19 to -0.22), which was consistent with our original risk estimate. No missing trials were imputed in the contour-enhanced funnel plot (Supplementary Figure S4).

## Discussion

In the current study, we systematically reviewed the literature to assess the efficacy of rTMS in MS patients with spasticity. The meta-analysis found that rTMS significantly improved spasticity in the early post-intervention period, although there was significant heterogeneity probably due to sample size or disease duration. Moreover, our results suggested no significant benefit of rTMS compared with sham control for spasticity reduction during the follow-up period, as well as no significant reduction in fatigue at the post-treatment evaluation.

Even though spasticity does not have exclusively negative aspects for the patient, since a paretic limb may allow the patient to continue to walk, stand, and move, there is a great variety of dramatic short- and long-term negative consequences on daily life activities (31-33). MS can have a fluctuating and often progressive course, making spasticity management more challenging. The relationship of rTMS and the two major symptoms of fatigue and spasticity in MS patients has also been the subject of an earlier meta-analysis, in which a non-significant association between rTMS treatment and early improvement of modified Ashworth scale scores was reported (pooled MD: -0.91; 95%CI: -1.91 to 0.09; P=0.07) (34). Although this provided valuable information, the overall quantitative synthetic data were based on a whole range of MS patients. Results of an early double-blind study by Nielsen et al. (24) pointed out that rTMS has beneficial effects against spasticity in patients with MS, which is inconsistent with this previously published meta-analysis. However, we found that the authors of the previous meta-analysis missed this important clinical study in the process of literature screening and data extraction for unknown reasons, which might bias the overall effect. We performed this study based on a notable assumption. Compared with previous systematic reviews, we focused exclusively on patients diagnosed with MS-related spasticity, as these patients are most likely to reveal a direct benefit of rTMS on spasm-related outcomes. Moreover, additional subgroup analyses according to different characteristics for each trial helped identify potential moderators affecting the efficacy of rTMS.

Despite the short-term positive effect of rTMS (P=0.004), patients randomized to stimulation versus control showed no trend toward a spasticity reduction at the follow-up visit 2 weeks later (P=0.32). Due to the limited number of studies and inconsistent follow-up period, this result should be interpreted carefully, and more evidence is required for its confirmation. In addition, no significant advantage of rTMS over sham has been found for improving fatigue, in line with Chen et al. (26) meta-analysis. In stratified analyses for the outcome of spasticity, a beneficial effect was observed for HF-rTMS application for SP MS in trials with the proportion of females >50% and in trials in which rTMS application was combined with rehabilitation training. It should be noted that no safety end-points are provided in our meta-analysis. Although Nielsen et al. (24) reported a case of irregular heartbeats 2 h after stimulation, no adverse effects were reported in other trials included in our meta-analysis, suggesting that rTMS treatment was safe and acceptable for most patients.

The beneficial effects of rTMS are likely to be mediated through changes in cortical excitability at the site of stimulation and by trans-synaptic changes at distant sites. Researchers speculate that rTMS induces lasting inhibitory or stimulatory aftereffects on corticospinal motor output, which may be due to long-term depression-like and long-term potentiation-like mechanisms, beyond the shifting in network excitability, activation of feedback loops, and activity-dependent metaplasticity phenomena (34,35). In this case, previous synaptic activities influence the level of reactivity to subsequent stimuli. Therefore, a priming stimulation may change various synaptic properties thus altering the effects of a subsequent plasticity-inducing event.

Despite the presence of some differences in the physiological effects, both the HF-rTMS and the iTBS protocols have the ability to increase the excitability of the motor cortex. It has been theorized that iTBS more closely mimics the brain's natural neuronal firing patterns and produces more robust changes in cortical excitability, thereby providing a more optimized cortical environment than the conventional HF-rTMS protocol for improved performance of the task (36). However, our analysis showed that HF-rTMS is non-inferior to iTBS for the treatment of spasticity in patients with MS. We speculated that the observed non-inferior effectiveness may be related to the differences of cumulative effects between iTBS and HF-rTMS. Further research including neural mechanisms is warranted. There is an emerging view that RR MS and SP MS are part of a disease continuum with an indistinct boundary, in which disability progression may result from neurodegeneration that is not linked to inflammation (37). This theory may explain the non-significant subgroup difference based on MS type. However, the possibility of chance findings cannot be ruled out due to the small number of studies for different subgroups and the small sample sizes of the included studies. Exercise rehabilitation is thought to facilitate activity-dependent neuroplasticity involving spinal pattern generators or motor pathways in the brain (38), and has been shown to modulate brain integrity/volume and functional connectivity (39). Additionally, direct central anti-inflammatory and pro-myelinating effects of exercise have been demonstrated in demyelinating models (40).

Through our comprehensive search strategy, stringent inclusion/exclusion criteria, and pre-specified subgroup analyses, we believe that we offer a valid summary of the published literature on rTMS in MS-related spasticity. As with any systematic review, the inherent limitations apply also to the present meta-analysis: lack of rigorous methodology and variable use of outcome measures, differences in the duration and severity of the disease among trials, and lack of robust studies with low bias, appropriate sample sizes, and power to detect expected differences. In addition, the follow-up periods varied between trials with most being immediately after treatment or up to 2 weeks. Only one trial reported long-term follow-up of up to 12 weeks, but this was restricted only for evaluation of patient-reported symptoms using subjective questionnaires (21); thus, the long-term response of rTMS therapy on MS-related spasticity could not be assessed. Also, for the secondary outcomes and the baseline features of interest, the numbers of studies included in quantitative analyses were extremely small; therefore, the results may be unreliable and need to be further tested in prospective studies.

## Conclusion

The result of this study suggested that real rTMS was superior to sham condition in reducing MS-related limb spasticity in the early post-intervention period. The effectiveness of rTMS on reducing spasticity at the short-term follow-up and fatigue was not significant. Taken together, our data supported recommendations to treat these patients with rTMS, but emphasized the need for large randomized trials before the result of this review can be generalized.

## Supplementary Material

Click here to view [pdf].
